# Perspectives of Italian Physicians and Patients in the Treatment of Otitis Externa: A Real-Life Study

**DOI:** 10.3390/jpm13071083

**Published:** 2023-06-29

**Authors:** Matteo Gelardi, Rossana Giancaspro, Massimo Landi, Luigi Santoiemma, Maddalena Balestra, Michele Cassano, Roberta Rizzo

**Affiliations:** 1 Unit of Otolaryngology, Department of Clinical and Experimental Medicine, University of Foggia, 71122 Foggia, Italy; matteo.gelardi@unifg.it (M.G.); michele.cassano@unifg.it (M.C.); 2 Paediatric National Healthcare System, 10135 Torino, Italy; landi@alma.it; 3 General Practitioner, Local Health Department Bari, 70026 Modugno, Italy; santoiemmaluigi@libero.it; 4 Pharmaceutical Chemist, 00143 Rome, Italy; m.balestra@fenixpharma.it; 5 Department of Chemical, Pharmaceutical and Agricultural Sciences, University of Ferrara, 44121 Ferrara, Italy; roberta.rizzo@unife.it

**Keywords:** otitis externa, otological preparations, ozone, liposome

## Abstract

(1) Background: Otitis externa (OE) is a common inflammatory disease of the external ear canal. Given the numerous manifestations of OE, the high rate of recurrence of the disease, as well as the emergence of resistance to antibiotics, therapeutic strategies are numerous and still not well standardized. The aim of this study was to investigate the patient journey of Italian patients suffering from OE, paying greater attention to new therapeutic options. (2) Methods: We conducted a national survey to evaluate the characteristics of patients affected by OE and to analyze the most-prescribed treatments. (3) Results: OE is a common, often relapsing disease that has several clinical manifestations. Prior to observation, the combination of corticosteroids and topical antibiotics was the most common therapeutic strategy. At the time of observation, new liposomal ozone-based preparations were the most-prescribed treatment. (4) Conclusions: This multi-center study investigated key aspects of the OE patient journey, highlighting the growing problem of antibiotic resistance. Restoring a correct ear microbiome is the therapeutic goal. In this context, new liposomal ozone-based drugs represent a promising therapeutic strategy.

## 1. Introduction

Otitis externa (OE) is a common inflammatory disease of the external ear canal, sometimes extending to parts of the outer ear such as the pinna or tragus, that can be either infectious or non-infectious. Approximately 10% of people develop OE during their lifetime such that it is responsible for more than 500,000 emergency department visits each year, with no gender predominance [1].

OE can be classified as acute (lasts less than 6 weeks), chronic (lasts more than 3 months), and necrotizing (malignant) [2]. Milder forms of acute OE are often short-lived isolated episodes; however, a considerable proportion of cases can persist for weeks or even months despite intensive treatment. Furthermore, once resolved, the latter forms show a significant risk of recurrence [3]. The clinical presentation of OE may vary depending on the stage or severity of the disease. The signs and symptoms of external ear canal inflammation include otalgia, otorrhea, hearing impairment, diffuse external ear canal erythema, and tenderness of the tragus, pinna, or both that is often intense and disproportionate to physical exam findings due to irritation of the highly sensitive periosteum underneath the thin dermis of the bony ear canal [4].

The most common cause of acute otitis externa is a bacterial infection caused by Pseudomonas aeruginosa (20–60% prevalence) and Staphylococcus aureus (10–70% prevalence), occurring as a polymicrobial infection [5]. Rarely, it may result from a fungal infection caused by Candida albicans or Aspergillus sp [6]. Fungal involvement is more common in chronic OE or after treatment of acute OE with topical or systemic antibiotics. Indeed, chronic OE has been shown to be associated with changes in the ear canal microbiome [7]. Moreover, common predisposing factors for OE include humidity or prolonged exposure to water, dermatologic disorders of the ear canal or conchal bowl (eczema, seborrhea, atopic dermatitis, psoriasis), anatomic abnormalities (narrow canal, exostoses), mechanical trauma, foreign bodies, or external devices (hearing aids, ear syringing) [8].

The therapeutic strategy for this disease is based on the use of systemic analgesics and topical antibiotics, such as aminoglycosides, polymyxin B, and quinolones. Moreover, topical medications include acetic acid, boric acid, and liposomal ozone [9]. The addition of oral antibiotics has been shown to have no significant benefit in the treatment of uncomplicated AOE limited to the ear canal in immunocompetent patients. Therefore, oral antibiotics should be limited to cases of extensive pathology outside the ear canal or in conditions such as immunocompromise, diabetes mellitus, or those that would not allow the use of topical treatment [10].

Despite the high prevalence of the disease, however, there are few data in the literature related to the most effective therapeutic strategy as well as regarding the therapeutic approaches most adopted by Italian otolaryngologists, general practitioners, and pediatricians. In fact, no previous studies have analyzed the therapeutic strategies currently adopted in a real-world Italian population from the point of view of both patients and physicians.

The aim of this survey was to investigate the demographic features of patients suffering from OE and the management strategies most used in clinical practice, paying greater attention to new therapeutic options.

## 2. Materials and Methods

We conducted a national survey recruiting 274 Italian medical doctors, including otolaryngologists, general practitioners, pediatricians, and internists. Physicians involved in this survey were asked to observe consecutive patients suffering from OE, collecting data regarding patients’ demographic characteristics, their signs and symptoms, the frequency of the episodes of OE, eventual risk factors, comorbidities, drug history and any treatment prescribed at the time of observation. Moreover, at the end of the survey, the participants were asked to respond to a SurveyMonkey survey aimed at evaluating their satisfaction with the use of new medications and the possible onset of side effects.

Since this survey was based on real-world practices, all physicians chose the pharmacological strategy freely according to their best practices.

All patients signed written informed consent.

## 3. Results

The survey was completed by 164 Italian medical doctors, including 130 otolaryngologists, 26 general practitioners, 7 pediatricians, and 1 internist, providing 4365 observational forms (average per physician: 16.4). Among the 4365 patients, 2186 (52.9%) were males and 1948 (47.1%) were females. Table 1 shows patient mean age, weight, height, and BMI.

In 2780 (68%) cases, the patients received a first diagnosis of OE. On the contrary, 1281 patients (32%) had already received a diagnosis of OE. Among them, 111 (11%) patients had episodic OE, while 895 (89%) suffered from recurrent OE. OE episodes most occurred in the summer months (2247 cases, 67%) and in patients aged 21 to 50 years (1629 cases, 45%).

**Figure 1:**
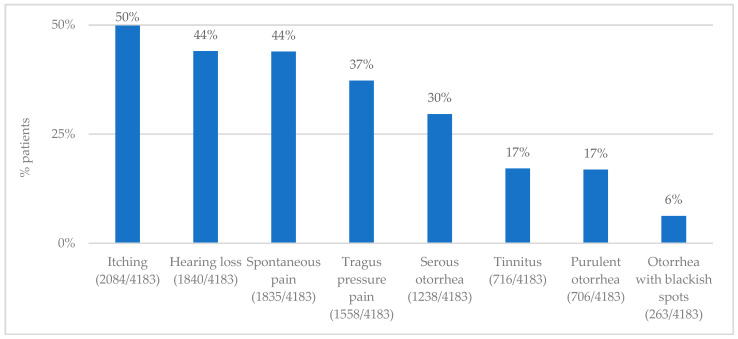
This figure shows symptoms complained of by patients.

**Figure 2:**
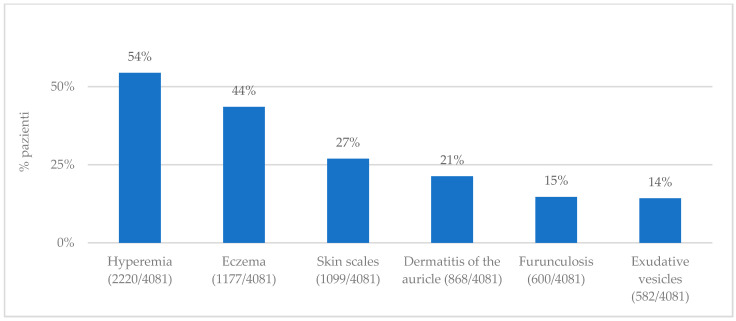
This figure shows signs found by medical doctors.

The patients complained of hearing loss, tinnitus, otorrhea (serous, purulent, with blackish spots), pain, tragus tenderness, and itching, variously associated, involving one (2776 patients, 68%) or both ears (1330 patients, 32%) (Figure 1). Moreover, the patients presented with varying degrees of ear canal eczema, furunculosis, hyperemia, exudative vesicles, skin scales, and dermatitis of the auricle (Figure 2).

**Figure 3:**
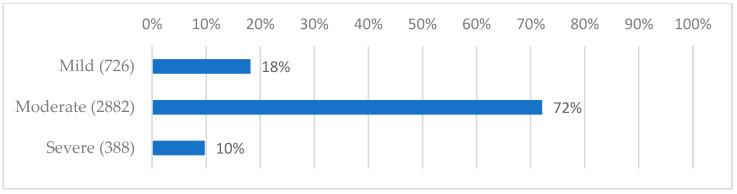
The average VAS score attributed to the well-being of the patients’ ear canals.

Figure 3 shows the average VAS score attributed to the well-being of the patients’ ear canals, which is then divided into groups according to whether the disturbance was considered mild, moderate, or severe.

As regards risk factors, 2076 patients had been exposed to hot humid environments, 1942 to mechanical trauma, 574 to removal maneuvers of the earwax plug, and 1584 to the use of cotton swab. Interestingly, most patients were not swimmers (2808), did not have a narrow ear canal (2856), and did not use hearing aids (2199) or earphones (2621). Additionally, 1060 patients (25%) suffered from different comorbidities, among which diabetes (608) and liver disease (133) were the most common. Most patients (3053, 76%) did not show sensitization of the skin of the ear canal. A bacterial infection was found in 1505 patients, while fungal colonization was found in only 499 of them. Infection extended to the auricle or tympanic membrane in 1034 and 930 cases, respectively.

Prior to observation, 1148 (29%) patients underwent treatment to resolve OE, including boric acid washes (35%), acetic acid washes (11%), topical corticosteroids (12%), topical antibiotics (21%), a combination of corticosteroids and topical antibiotics (40%), topical antifungals (10%), and oral antibiotics (22%). The adoption of preventive measures (including keeping the ear dry, treating eczema with topical corticosteroids, applying acetic acid or aluminum acetate) was considered in only 732 patients. The use of ozone-based preparations had only been considered in 631 cases.

**Figure 4:**
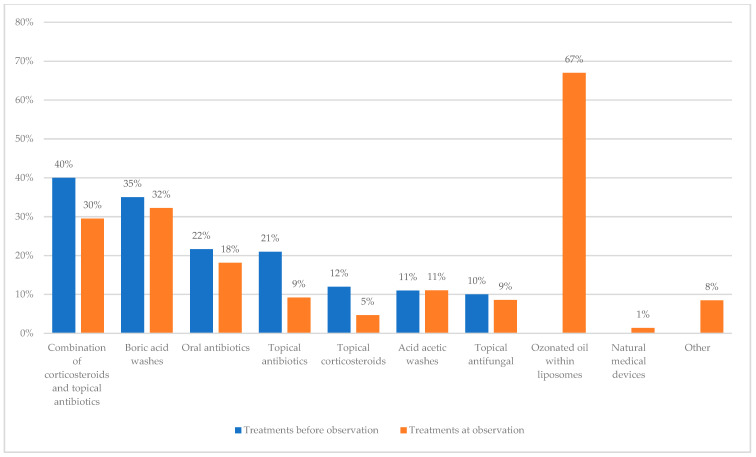
This figure summarizes the prescribed treatments before observation (blue) and at the time of observation (orange).

At the time of observation, 3791 patients (95%) were prescribed treatments to resolve OE, while 195 (5%) patients did not receive any treatment. This change of course in the therapeutic strategy depended on greater awareness on the part of the doctors who joined the project and were made aware of the importance of treating OE correctly to reduce the risk of chronicization or evolution to malignant OE. Figure 4 summarizes the prescribed treatments as well as the comparison between the treatments prescribed before observation and at the time of observation. Ozone-based preparations were prescribed in 1477 cases (57%).

At the end of the survey, the participants who were prescribed ozone-based preparations, eventually associated with other treatments, found an improvement (reduction in VAS score after treatment by at least 3 points compared to VAS score at observation) in OE in 40% of cases and a resolution (VAS = 0) in 60% of cases.

Since this survey was based on real-world practice, all physicians chose the pharmacological strategy freely according to their best practices.

## 4. Discussion

OE is a common multifactorial disease presenting with otalgia, tenderness, diffuse ear canal edema, and otorrhea. These symptoms can also cause severe discomfort and sleep disturbances, resulting in many health care visits [11]. According to practice guidelines, the treatment for uncomplicated AOE includes topical antimicrobial agents with or without anti-inflammatory drugs, risk factor avoidance, and pain management, while the use of systemic antibiotics is strongly discouraged as an initial therapy [12]. As a matter of fact, topical antibiotics are the preferred first-line treatment as they achieve high local concentrations in the infected area, with a low rate of adverse events (AEs).

Moreover, the addition of corticosteroids to ototopical antibiotic treatment has been shown to enhance the resolution of the inflammatory response and improve associated symptoms due to their anti-inflammatory, antipruritic, and vasoconstrictive properties [13].

However, by virtue of the numerous manifestations of OE and the recurrence of the disease, as well as the onset of antibiotic resistance, the development of new therapeutic tools with rapid and long-lasting efficacy appears of fundamental importance. Nevertheless, to develop novel medications for OE, several prerequisites must be met, including addressing important issues such as sufficient coverage of pathogenic microorganisms and lack of bacterial resistance, allergic reactions, and ototoxicity [14]. Indeed, although ototopical antibiotics have traditionally been the mainstay treatment of OE, there is widespread concern about the development of antibiotic resistance leading to disease recurrence. Furthermore, the fungal etiology, which requires a different treatment, should not be underestimated [15]. Among other things, otomycoses are often associated with perforations of the tympanic membrane. The involvement of the tympanic membrane could be the result of fungal inoculation in the more medial aspects of the external canal or the direct extension of the disease from the adjacent skin. Tympanic membrane perforations could be a complication of otomycosis, directly associated with fungal thrombosis of the adjacent blood vessels and subsequent avascular necrosis [16]. The possible presence of this complication could cause many treatment difficulties, since the seepage of topical antifungal ear drops to the middle ear cavity may cause intense pain and a severe burning sensation and, above all, some antifungal agents may be ototoxic. However, the problem of ototoxicity does not only apply to antifungal agents, but also other common antibiotic-based ear drops and acidic ear irrigation solutions. Indeed, there is great evidence that drops containing aminoglycosides can cause ototoxicity, especially insidious vestibular ototoxicity [17]. Moreover, the use of quinolone ear drops has been linked to an increased risk of tympanic membrane perforation due to the cytopathic effects on tympanic membrane fibroblasts [18].

For this reason, with the intention of reducing side effects, numerous studies have aimed to identify the relative ototoxicity of otological drops commonly used not only for OE, but also for otorrhea in the presence of tympanostomy tubes or tympanic membrane perforations [19].

Based on this background, this study aimed to provide real-world evidence for all aspects of a patient’s journey to understand the characteristics of OE patients, current clinical and therapeutic approaches, and outcomes in Italy.

The first noteworthy aspect highlighted by this survey is that the participants were mainly otolaryngologists, precisely because OE patients tend to rely on the care of ENT specialists rather than their general practitioners due to the severity and rate of disease recurrence. This led to a discrepancy between what was found in the survey and what was described in the literature in relation to the epidemiological characteristics of OE. In fact, this pathology tends to affect mainly children, with a peak between 7 and 14 years. The population in question, on the other hand, had a higher average age, approximately 45 years of age, probably precisely because only seven pediatricians joined the survey, with a consequent increase in the average age of the population [2]. No discrepancy was found regarding the gender of the patients since the survey confirmed that OE does not have a gender predominance. Among the patients investigated, 32% had already received a diagnosis of OE and suffered from a relapsing or, in most cases, recurrent form of OE. The latter form would seem to be related to an alteration of the ear microbiome. Indeed, it has been shown that the healthy ear core microbiome includes *Staphylococcus capitis* and *S. capitis/caprae*, while the diseased ear core is composed of known bacterial and fungal pathogens, such as *Aspergillus* sp., *Candida* sp., *Pseudomonas aeruginosa*, *S. aureus*, and *Corynebacterium jeikeium*. This awareness underscores the importance of targeting therapies not only to eliminate pathogens, but also to restore a healthy microbiome in order to improve treatment outcome and limit inappropriate antibacterial treatments, thereby improving overall antimicrobial stewardship [20]. The higher incidence of OE found in the summer months also highlights the importance of the ear microbiome. Indeed, OE displays a characteristic seasonal variation, with a greater disease burden in warmer months. Predisposing factors for OE include long exposure to moisture, with an increased risk of maceration and earwax loss. Contact with contaminated water and increased water temperature favors the rapid growth of *P. aeruginosa* and *S. aureus*, such that the proportional incidence of *P. aeruginosa* related to the number of rainy days has been observed [21]. Moreover, hot–humid environments, to which half of the patients examined had been exposed, predispose the development of biofilm, which is now counted among the causes of chronicity of OE [22]. Even ear irrigation for the removal of earwax plugs may be related to the development of OE, so much so that more than half of the patients examined had undergone this procedure. This is related both to the use of instruments contaminated by *P. aeruginosa* and to residual humidity. However, there is no evidence that OE can follow ear irrigation if the procedure is performed using a clean technique [23]. Interestingly, most of the patients did not usually go to swimming pools, whose poor-quality water is often associated with the development of otitis. These data probably reflect the small number of pediatricians who took part in the survey, since children are the ones who most commonly go to public swimming pools [24]. In addition to the pathogens of contaminated swimming pool water, it is noteworthy that typical pathogens of marine environments, such as *Vibrio alginolyticus*, can also be responsible for the onset of OE. Indeed, this Gram-negative rod bacterium can be responsible for opportunistic infections in humans, including ear infections, which can be difficult to diagnose and treat [25]. Therefore, an ear infection caused by this pathogen should be suspected in any patient who develops ear infections after participating in aquatic activities, such as swimming, diving, fishing, or boating, or during any exposure to a marine environment or animal.

An other remarkable aspect shown in the results is that most of the patients had suffered scratch trauma or had used cotton-tip applicators: both situations result in damage to the epithelium, loss of protective wax, and accumulation of moisture, leading to a higher pH and bacterial growth [26,27]. Among the comorbidities, the most frequent was represented by diabetes, which is known to be an important risk factor for various bacterial infections and, above all, for the evolution of OE towards the malignant form [28]. Malignant OE is an aggressive type of OE associated with extensive inflammation and osteomyelitis of the skull base bone, usually affecting individuals with diabetes mellitus. The most involved microorganism is represented by Pseudomonas species [29]. To optimize clinical outcome, malignant OE requires rigorous antimicrobial treatment and close monitoring of patients with pre-existing comorbidities, facial nerve paralysis, extensive disease, and markedly elevated inflammatory markers [30]. However, in non-responsive patients after a period of at least six weeks of conventional treatment, surgery should be considered, including local debridement, canal wall up (CWU) mastoidectomy, or canal wall down (CWD) mastoidectomy, according to the extent of the infection [31,32,33].

As shown in the results, prior to observation, only 29% of patients had been treated to resolve OE, mostly with oral or topic antibiotics, eventually with boric acid or acetic acid washes, and only a minimal percentage with topical corticosteroids. Moreover, preventive treatments were recommended only in 18% of cases.

On the contrary, at the time of observation, medical doctors that endorsed the survey, aware of the importance of correctly treating OE in order to restore the ear microbiome and reduce OE recurrence, prescribed to most patients (95%) medical treatments aimed at resolving the disease. Interestingly, in 57% of cases, new liposomal ozone-based medications that had never been prescribed before the observation were prescribed at the time of observation.

As a matter of fact, ozone is the most powerful oxidizing agent found in nature, commonly known for its antiseptic and anti-inflammatory properties. Ozone acts selectively on microbial cells thanks to direct antioxidant action, peroxidation of membranes, and degradation of DNA, RNA, and plasmids, as well as alteration of vital proteins and enzymes. In fact, in the presence of ozone, microorganism lipoproteins generate products of lipid oxidation, which in turn induce higher amounts of H_2_O_2_ from phagocytes, resulting in better bacteriostatic and bactericidal activity [9].

Furthermore, it promotes the release of growth factors promoting wound healing, such as PDGF and TGF-β, and collagen synthesis. However, ozone alone cannot be used in otological preparations, as it would cause irritation of the mucous membranes of the ear [34]. On the contrary, the combination with liposomes, which are spherical vesicular structures in aqueous suspension made up of phospholipids organized in a bilayer, stabilizes ozone and makes it tolerated by the ear surface. In particular, a nanoemulsion with ozonated oil within liposomes not only favors transcutaneous absorption, protection, and in situ transport of the encapsulated active principle as well as skin nourishment and emollience, but also inhibits the growth of bacteria and fungi without creating antibiotic resistance. Indeed, antibiotics act on a specific target of a microorganism, which can develop defense and resistance mechanisms. Contrarily, ozone has been shown to prevent and eradicate biofilm [35,36]. Moreover, ozone can also control interaction with viral capsid, inducing the peroxidation of glycoproteins and lipids, leading to the weakness of lipid-coated viruses and to a drastic reduction in the viral load, with mild intracellular and mitochondrial oxidative-stress-mediated signaling able to trigger an antioxidant, anti-inflammatory, anti-thrombogenic response [37].

Precisely by virtue of the growing data present in the literature relating to the properties of new liposomal ozone-based drugs in the mainly ophthalmological field, as well as the need to define new therapeutic strategies aimed at resolving the various forms of OE without favoring the onset of antibiotic resistance, the participants of the survey prescribed these new treatments with good therapeutic results.

## 5. Conclusions

This real-world multicenter study highlights the main aspects of the OE patient journey while also emphasizing the increasingly widespread problem of antibiotic resistance, consistent with the recent literature, and the need to re-establish a correct microbiome to effectively treat infectious diseases.

This survey underlines that nowadays new treatments are available for OE with the precise aim of bypassing the common antibiotic mechanisms and reducing antibiotic resistance. In this context, studies on a larger scale on the efficacy of oxidizing agents and, in particular, on liposomal ozone-based medications in the treatment of OE would be useful to standardize the therapeutic approaches and reduce the risk of recurrence.

## Figures and Tables

**Table 1:** Patient demographic characteristics.

	Male	Female
Sex, number (%)	2186 (52.9)	1948 (47.1)
Age, years (DS)	45.9 ± 20.8	43.8 ± 20.1
Weight, Kg (DS)	74.3 ± 15.4	62.7 ± 14.7
Height, cm (DS)	170.7 ± 14.2	162.3 ± 12.7

## Data Availability

The data that support the findings of this study are available from the corresponding author, R.G., upon reasonable request.
